# Smoking among adolescents in Jordan: Prevalence, associated factors, and its relation to mental health outcomes

**DOI:** 10.1371/journal.pone.0342653

**Published:** 2026-02-26

**Authors:** Sara Abu Khudair, Yousef Khader, Mohannad Al Nsour, Eizaburo Tanaka

**Affiliations:** 1 Eastern Mediterranean Public Health Network (EMPHNET), Amman, Jordan; 2 Department of Community Medicine, Public Health and Family Medicine, Jordan University of Science and Technology, Irbid, Jordan; 3 Center for Research on Counseling and Support Services, The University of Tokyo, Tokyo, Japan; The University of Jordan, JORDAN

## Abstract

**Purpose:**

This study aimed to explore the prevalence of various forms of smoking among adolescents in Jordan, identify associated factors, and examine their relationships with mental and psychosocial problems.

**Methods:**

Data were obtained from a large-scale, nationally representative, school-based survey conducted between December 2022 and April 2023 among children and adolescents aged 8–18 years in both host and refugee populations in Jordan. A multi-stage stratified cluster sampling design was employed to ensure national representativeness. The study utilized internationally recognized and validated tools that assessed several mental and psychosocial problems. The present analysis was restricted to adolescents only.

**Results:**

Among the 4,407 adolescents included in the study, the most prevalent form of tobacco use was waterpipe (shisha) at 19.2%, followed by e-cigarettes (15.1%) and cigarettes (9.8%). In multivariate analysis, smoking odds increased significantly with age. Notably, compared with 12-year-olds, adolescents aged 18 had significantly higher odds of smoking cigarettes or waterpipe (OR = 5.9, 95% CI 3.8–9.2) and using e-cigarettes (OR = 4.5, 95% CI 2.7–7.4). Adolescents whose mothers had less than a diploma had lower odds of e-cigarette use (OR = 0.8, 95% CI 0.6–0.9). Compared with adolescents in central Jordan, those in the northern region had significantly lower odds of both cigarette/waterpipe use (OR = 0.8, 95% CI 0.6–0.9) and e-cigarette use (OR = 0.7, 95% CI 0.6–0.9). Palestinian camp refugees were significantly less likely than Jordanians to smoke cigarettes or waterpipe (OR = 0.4, 95% CI 0.2–0.9) but did not differ significantly in e-cigarette use. Smoking cigarettes or waterpipe was significantly associated with higher levels of several mental and psychosocial problems symptoms, including separation anxiety, emotional and behavioral difficulties (emotional symptoms, conduct problems, hyperactivity, relationship problems with peers, lower sociability, general behavioral difficulties), and PTSD, along with a higher risk of problematic Internet use. Also, smoking cigarettes or waterpipe was significantly associated with poorer quality of life across all dimensions (overall, physical health, psychosocial health, emotional functioning, social functioning, and school functioning). Adolescents who reported using e-cigarettes had significantly higher conduct problems, hyperactivity symptoms, and total difficulties scores, as well as lower prosocial behavior and poorer school functioning.

**Conclusion:**

Adolescent smoking in Jordan remains a pressing public health issue, with waterpipe use emerging as the most common form and increasing with age across different nationalities. Smoking, whether in the form of cigarettes, waterpipe, or e-cigarettes, was associated with increased vulnerability to a range of mental health issues and diminished health-related quality of life. Addressing this issue requires a multifaceted and evidence-based approach, including developing school-based prevention and control programs, incorporating social competence and social influence curricula, enforcing existing tobacco laws, and introducing updated regulations in response to emerging trends and evidence, particularly targeting flavored products. Furthermore, prevention and control strategies need to implement targeted interventions that address both the psychosocial roots of smoking and its consequences.

## Introduction

Smoking remains a leading risk factor for non-communicable diseases, including cancer and cardiovascular diseases. According to the 2015 Global Burden of Disease (GBD) study [1], 11.5% of global deaths were attributed to smoking worldwide. While smoking prevalence rates are declining worldwide, the pace of reduction varies across regions. In the Eastern Mediterranean Region (EMR), the decline is slow, making it highly unlikely that the region will achieve the World Health Organization (WHO) target of a 30% relative reduction by 2025 [2].

Jordan, in particular, exhibits high rates of tobacco smoking compared to many countries in the region and globally. In response, the Jordanian government has been proactive in advancing tobacco prevention and control by providing publicly accessible cessation services, launching mass media campaigns, and administering public health policies [3]. However, challenges persist, particularly in maintaining consistent progress, enforcing policies, and updating them based on emerging smoking trends among different age groups. For instance, Jordan’s performance in implementing the MPOWER measures was ranked 83rd globally in 2018, with a modest improvement in scores of only 8.3% from 2008 to 2018 [4].

The most recent national study among adults in Jordan reported crude prevalence rates of 32.0% for current cigarette smoking, 11.0% for current waterpipe smoking, and 2.8% for dual smoking [5]. The corresponding age-standardized prevalence rates were 25.5% for cigarette smoking, 12.0% for waterpipe smoking, and 2.8% for dual smoking [5]. The 2019 STEPwise approach to noncommunicable disease surveillance (STEPs) survey underscored the severity of the smoking issue among adults, reporting that 41% of the surveyed population currently smoked conventional tobacco products (daily and non-daily), with a higher prevalence in males (65.3%) than females (16.4%) [6]. In Jordan, smoking among adults predominantly involves conventional tobacco products, primarily manufactured cigarettes and waterpipes. STEPS 2019 findings suggest that waterpipe constitutes a relatively larger share of tobacco use among women compared with men, whereas the use of emerging tobacco products, such as e-cigarettes and vaping devices, is more common among younger adults [6].

However, more recent data on smoking habits and characteristics among the younger population in Jordan remain limited. Emerging evidence suggests that the COVID-19 pandemic intensified mental health challenges among youth and contributed to shifts in risky behaviors, including a rise in smoking rates during the pandemic period [7]. This trend raises concerns about sustained smoking behaviors and underscores the importance of updated and representative national data on youth smoking patterns. It is also important to note that adolescent smoking is shaped by several established factors, including peer and parental smoking, low parental monitoring, low parental education, and engaging in other health-risk behaviors [8]. In addition, cultural norms and the type of product influence use, where flavored products such as e-cigarettes and shisha could be perceived as less harmful [9] and have made uptake increasingly acceptable among females in certain communities [10].

In Jordan, recent findings from a nationwide survey highlighted major mental health and psychosocial challenges among adolescents. For example, nearly 35% of adolescents had poor overall health-related quality of life, and 19.7% had abnormal levels of emotional and behavioral difficulties [11]. The relationship between smoking and mental health issues among adolescents is complex and multifaceted, but it is established in the literature, and it is important to explore it to understand the behavioral patterns linked to smoking and to help in the development of effective and targeted interventions [7,12].

Beyond the challenges in implementing and updating smoking policies in Jordan, there is a notable lack of recent, nationally representative data across a broad age range on adolescent smoking. Additionally, there remains a critical need for more detailed studies on youth smoking behaviors, particularly those that examine various forms of smoking and their associations with important factors such as mental health. Such results are important given Jordan’s youthful population structure, global evidence that smoking during adolescence increases the likelihood of smoking in adulthood [13], the early average age of smoking initiation in Jordan (17.1 years among adult men in 2019) [6], and the well-established link between adolescent smoking and mental health issues [7]. More updated and detailed data on adolescent smoking patterns across products, age groups, and nationalities, including refugees and their associations with mental health outcomes (a major concern among young people) are therefore crucial to inform effective, targeted, and equitable tobacco-control policies and prevention programs in Jordan. Thus, this study aimed to explore the prevalence of various forms of smoking among adolescents in Jordan, identify associated factors, and examine their relationship with mental and psychosocial problems.

## Methods

### Study design

Data were obtained from a large-scale, nationally representative, school-based survey conducted between December 2022 and April 2023 among children and adolescents aged 8–18 years in both host and refugee populations in Jordan. The study included Jordanians and those of other nationalities and groups, such as Syrian and Palestinian refugees. The national study included two versions: a proxy parent version for parents of students in grades 3–6 and a self-report version for students in grades 7–12. The expected age of students in the parent version ranged from 8 to 11 years (children group) and 12–18 years for the self-report version (adolescents group), based on the educational ladder of the MoE. The present analysis focuses exclusively on the adolescent group.

### Sampling

The study utilized a multi-stage stratified cluster sampling technique to select a nationally representative sample. The sample covered the main school sectors in Jordan in basic and secondary education, including the Ministry of Education (MoE), the private sector, and the United Nations Relief and Works Agency (UNRWA) for Palestine refugee schools in Jordan. The school population was stratified into different explicit strata. The first level of stratification is governing authority (MoE, private, and UNRWA). Within these strata, there is implicit stratification by region (North, South, and Central) and governorate (12 governorates). Within the MoE stratum, the second level of explicit stratification is the refugee context (Regular MoE schools and Syrian second-shift schools). Regular MoE schools and MoE Syrian second-shift schools are further stratified by gender (boys, girls, and mixed). Because of its small size, half of the Zaatari refugee camp schools were selected and gender balanced. A list of all schools was obtained from the MoE. Schools were ordered according to the number of students. A systematic sample was selected from each stratum. All classes (grades 3–12) in the selected schools were included, and each class was defined as a unit for data collection. A systematic sample of 30% of students in each class was selected. Further details are described in the main study [11].

The sample size for the main study involving children and adolescents was calculated assuming a 25% prevalence of common mental disorders (e.g., depression) among children and adolescents, a 95% confidence level, and a margin of error of 4%, yielding an estimated minimum of 1,801 participants. To account for clustering, a design effect of 2 was applied, and, considering an anticipated 70% completion rate, the required sample size was increased accordingly. To ensure sufficient power for subgroup analyses, the number of children and adolescents invited to participate in the survey was further expanded to 9,000.

The inclusion criteria for the current analysis encompassed only adolescents \ who filled out the study questionnaire and were enrolled in formal education settings (schools) and non-formal education centers in Jordan. Adolescents were asked to provide informed consent to participate by themselves. Exclusion criteria included students with debilitating cognitive impairments that could interfere with understanding the questionnaire items.

### Study tools

The study questionnaire focused on three main themes: (1) School characteristics, socio-demographic details, and health status; (2) instruments and questions assessing mental health and psychological problems; and (3) instruments and questions related to health behaviors, such as smoking. The selected instruments were translated into Arabic using a forward-backward method and culturally adapted when an Arabic version was unavailable. Two bilingual experts independently performed forward translations, and a preliminary Arabic version was created through a consensus meeting. This version was then backtranslated into English by two other bilingual experts. An expert committee, including translators, clinical health scientists, and an epidemiologist, reviewed the original and translated versions, resulting in a pre-final Arabic tool. Special attention was given to item clarity to ensure consistent cognitive understanding among respondents.

Participation in the survey was entirely voluntary and anonymous. Respondents had the option to skip any questions or discontinue the survey at any point if they felt uncomfortable. Special care was taken to ensure the clarity of the survey items, aiming to facilitate consistent cognitive processing among all participants. Also, a pilot test was conducted to evaluate the survey’s content and the data collection process, and regular quality assurance measures were implemented throughout the data entry process to ensure data quality. A more detailed explanation of the study’s methodology, including scoring, sampling, and other details, is described in the main study [11].

In the current study, multiple instruments were used to assess mental health and psychological problems,symptoms, and others. Additional details on each tool can be found in the main study [11]. The Family Affluence Scale (FAS)-III [14] was used to assess the socioeconomic status. It is a six-item self-administered scale measuring family material assets and living standards, including items such as car ownership, bedroom occupancy, number of computers, dishwashers, bathrooms, and family holidays. Higher scores indicate higher socioeconomic status. The Revised Child Anxiety and Depression Scale (RCADS) [15] was used to measure symptoms of anxiety and depression. RCADS consists of 47 items rated on a four-point Likert scale and yields six subscales: separation anxiety disorder, social phobia, generalized anxiety disorder, panic disorder, obsessive–compulsive disorder, and major depressive disorder. Two composite scores are generated: a Total Anxiety Score (sum of the five anxiety subscales) and a Total Internalizing Score (sum of all six subscales). Raw scores were converted to T-scores using standardized norms, with T-scores ≥70 indicating clinically significant symptoms. The Strengths and Difficulties Questionnaire (SDQ) [16] was used to assess emotional and behavioural problems. The SDQ consists of 25 items rated on a three-point scale and is divided into five domains: emotional symptoms, conduct problems, hyperactivity, peer problems, and prosocial behavior. A Total Difficulties Score is calculated by summing the first four subscales (20 items), with higher scores indicating greater psychosocial difficulties.

The Children’s Impact of Event Scale- 13 (CRIES-13) [17] was used to assess symptoms for post-traumatic stress disorders. It is a 13-item self-report instrument measuring three PTSD symptom domains: intrusion (4 items), avoidance (4 items), and arousal (5 items). Items are scored on a four-point scale and summed to produce a total PTSD score, with a cut-off score of 30 indicating high PTSD symptom levels.

The Pediatric Quality of Life Inventory (PedsQL) [18] was used to measure the health-related quality of life (HRQoL) in children and adolescents. It is a 23-item instrument measuring four domains: physical functioning (8 items), emotional functioning (5 items), social functioning (5 items), and school functioning (5 items). Higher scores indicate a better quality of life. The Problematic Internet Use Questionnaire (PIUQ-6) [19], was used to assess problematic internet use. The PIUQ-6 contains six items covering three domains: obsession, neglect, and control disorder. Items are rated on a five-point Likert scale and summed to generate a total score, with higher scores indicating a greater risk of problematic internet use.

As for smoking, three questions were asked about the use of cigarettes, e-cigarettes, and waterpipe (shisha), each independently, and with three response options: “every day,” “some days,” and “no”. Respondents were categorized as smokers if they answered, “every day” or “some days” for each smoking product, and non-smokers if they answered “no”.

### Statistical analysis

Data analysis was conducted using IBM SPSS 24. Frequencies and percentages were used to describe categorical variables. Logistic regression models were used to assess the associations between smoking (dependent variable) and participants’ demographic characteristics (independent variables). The goodness of fit of the logistic regression models was evaluated using the Hosmer–Lemeshow test. Separate General Linear Models (GLM) were employed to evaluate the association between smoking (cigarettes or waterpipe and e-cigarettes) as independent variables and scores on mental and psychosocial problem scales (dependent variables). For each mental and psychosocial problem scale, the effect of smoking was adjusted for school classification, type, gender, region, nationality, parents’ marital status, mother and father education, working status, and family income source. Statistical significance was determined at P < 0.05.

### Ethics approval and consent to participate

This survey followed the “Declaration of Helsinki” and other relevant local ethical guidelines for medical and health research involving human subjects. Written informed consent to participate was obtained from students in grades 7–12. Students first received both verbal and written explanations detailing the study’s objectives, potential risks and benefits, confidentiality safeguards, the anonymous nature of participation (no names collected), and their right to withdraw at any time without consequences or explanations. All participants were informed that if participation causes any stress or discomfort, they can withdraw from the study without any ensuing penalties or conditions. Also, participants were informed that all data will be kept in a safe, locked cabinet with only limited access by the project team. A written consent was then confirmed through a question within the survey assessing willingness to participate. All consent procedures were completed prior to the commencement of data collection. To minimize missing data, data collectors encouraged participants to complete all relevant items in the survey. To reduce bias and socially desirable responses, no personal identifiers were collected, and the anonymity and confidentiality of responses were clearly emphasized during data collection. For adolescents completing the self-administered questionnaire, data collectors ensured adequate spacing between participants to safeguard privacy and prevent peer influence. Ethical approvals were obtained from the Institutional Research Committees at Jordan University of Science and Technology (Ref. 11/151/2022) and Jordan Ministry of Health (Ref. MBA/16289).

## Results

### Adolescents’ characteristics

This study included 4,407 adolescents living in Jordan. The majority were Jordanians (66.2%), followed by out-of-camp Syrian refugees (16.8%). Nearly half were female (55.5%), and 44.5% were male. Most adolescents (63.8%) attended public schools. The majority resided in the central (51.1%) and northern (38.7%) regions of Jordan, with only 10.2% living in the southern region. About 78.6% of participants reported that their fathers were employed, while 25.0% indicated that their mothers were working. About half (55.5%) of the adolescents belonged to moderately affluent families, and 28.3% reported having had a confirmed COVID-19 infection.

### Prevalence of smoking in adolescents

The prevalence rates of cigarette smoking, waterpipe smoking, and e-cigarette smoking among adolescents were 9.8%, 19.2%, and 15.1%, respectively. The rates were higher among boys than girls across all smoking products, including cigarettes (12.2% vs. 8.0%), waterpipe (21.5% vs. 17.3%), and e-cigarettes (18.5% vs. 12.5%), as shown in Table 1.

**Table 1:** Prevalence of smoking various tobacco products among adolescents (aged 12–18 years) by gender.

Tobacco product ^a^	Adolescents (12–18 years)		
	Male	Female	Total
**Cigarettes**	12.2%	8.0%	9.8%
**Waterpipe**	21.5%	17.3%	19.2%
**E-cigarette**	18.5%	12.5%	15.1%

^a^For each product, a user was defined as a daily or occasional user of the product. Categories are not mutually exclusive.

Smoking waterpipe was the most common smoking method among Jordanians, Syrian camp refugees, and Syrian urban refugees (18.8% to 19.5%), followed by e-cigarettes (14.3% to 15.6%) and cigarettes (8.7% to 11.9%), as shown in Table 2. However, among Palestinian camp refugees, e-cigarettes were the most used, at 9.6%, closely followed by cigarettes and waterpipe at 6.8% each.

**Table 2:** Prevalence of smoking various tobacco products among adolescents (aged 12–18 years) by nationality.

Smoking product ^a^	Jordanian	Syrian camp refugees	Syrian urban refugees	Palestinian camp refugees	Others
**Cigarettes**	9.8%	11.9%	8.7%	6.8%	10.3%
**Waterpipe**	19.0%	19.5%	18.8%	6.8%	29.0%
**E-cigarette**	15.3%	15.6%	14.3%	9.6%	18.1%

^**a**^ For each product, a user was defined as a daily or occasional user of the product. Categories are not mutually exclusive.

The sex and age-specific prevalence rates of smoking among adolescents are shown in Table 3. The prevalence of smoking, including cigarette smoking, waterpipe smoking, dual smoking (cigarettes and waterpipes), cigarette or waterpipe only, as well as the use of e-cigarettes, appears to rise as adolescents get older, especially in males. Male adolescents aged 18 years had the highest rates of smoking different products and forms, including smoking waterpipe (23.8%), engaging in dual smoking behavior (cigarettes and waterpipe) (19.0%), using either cigarettes or waterpipe (46.0%), and using e-cigarettes (33.3%).

**Table 3:** Smoking percentages among adolescents by smoking products and patterns, sex, and age ^a.^

Sex/ Age	N	Cigarettes only		Waterpipe only		Dual smoking (cigarettes and waterpipe)		Any cigarette or waterpipe use		E-cigarettes	
		n	%	n	%	n	%	n	%	n	%
**Boys**											
**12**	201	4	2.0	18	9.0	11	5.5	33	16.4	21	10.4
**13**	220	4	1.8	15	6.8	12	5.5	31	14.1	34	15.5
**14**	269	4	1.5	40	14.9	18	6.7	62	23.0	45	16.7
**15**	448	18	4.0	55	12.3	47	10.5	120	26.8	90	20.1
**16**	363	13	3.6	43	11.8	42	11.6	98	27.0	75	20.7
**17**	237	7	3.0	35	14.8	25	10.5	67	28.3	48	20.3
**18**	63	2	3.2	15	23.8	12	19.0	29	46.0	21	33.3
**Girls**											
**12**	299	5	1.7	21	7.0	16	5.4	42	14.0	33	11.0
**13**	397	9	2.3	40	10.1	20	5.0	69	17.4	39	9.8
**14**	491	10	2.0	62	12.6	35	7.1	107	21.8	68	13.8
**15**	469	14	3.0	57	12.2	25	5.3	96	20.5	62	13.2
**16**	393	9	2.3	50	12.7	27	6.9	86	21.9	56	14.2
**17**	187	1	0.5	22	11.8	9	4.8	32	17.1	21	11.2
**18**	63	0	0.0	10	15.9	4	6.3	14	22.2	8	12.7
**Total**											
**12**	500	9	1.8	39	7.8	27	5.4	75	15.0	54	10.8
**13**	617	13	2.1	55	8.9	32	5.2	100	16.2	73	11.8
**14**	760	14	1.8	102	13.4	53	7.0	169	22.2	113	14.9
**15**	917	32	3.5	112	12.2	72	7.9	216	23.6	152	16.6
**16**	756	22	2.9	93	12.3	69	9.1	184	24.3	131	17.3
**17**	424	8	1.9	57	13.4	34	8.0	99	23.3	69	16.3
**18**	126	2	1.6	25	19.8	16	12.7	43	34.1	29	23.0

^a^Cigarettes only: smoking cigarettes exclusively and not smoking waterpipes; Waterpipe only: smoking waterpipe exclusively and not smoking cigarettes; Dual smoking: smoking both cigarettes and waterpipe; Cigarette or Waterpipe: smoking either cigarettes or waterpipes, or both; E-cigarettes: using e-cigarettes, regardless of other smoking methods.

### Factors Associated with Adolescents’ Smoking Behavior

The multivariate analysis presented in Table 4 shows an increase in the odds of smoking cigarettes or waterpipe, and e-cigarettes (dependent variables) with increasing age. Compared with 12-year-olds, adolescents aged 18 had significantly higher odds of smoking cigarettes or waterpipe (OR = 5.9, 95% CI 3.8–9.2) and using e-cigarettes (OR = 4.5, 95% CI 2.7–7.4). After adjusting for relevant variables, the effect of sex on smoking rates diminished, indicating that gender was no longer a significant factor in smoking behavior differences.

**Table 4:** Multivariate analysis of factors associated with the adolescents’ use of smoking products.

Variable ^a^	Cigarettes or waterpipe ^b^				E-cigarettes ^c^			
	OR	95% CI		P-value	OR	95% CI		P-value
**Age (years)**								
12	1.0				1.0			
13	2.0	1.4	2.7	<0.001	1.9	1.3	2.7	0.001
14	3.1	2.3	4.1	<0.001	2.6	1.9	3.7	<0.001
15	3.2	2.4	4.2	<0.001	2.7	2.0	3.8	<0.001
16	3.5	2.6	4.7	<0.001	3.0	2.2	4.2	<0.001
17	3.3	2.4	4.6	<0.001	2.8	1.9	4.0	<0.001
18	5.9	3.8	9.2	<0.001	4.5	2.7	7.4	<0.001
**Gender (female vs. male)**	1.0	0.7	1.5	0.954	1.0	0.7	1.6	0.839
**Mother Education (Less than diploma vs. Diploma or higher education)**	0.9	0.8	1.1	0.251	0.8	0.6	0.9	0.007
**Region**								
Middle	1.0				1.0			
South	1.0	0.8	1.3	0.785	1.1	0.8	1.5	0.604
North	0.8	0.6	0.9	0.008	0.7	0.6	0.9	0.008
**Nationality**								
Jordanian	1.0				1.0			
Syrian living in a camp	1.0	0.7	1.3	0.876	1.0	0.7	1.4	0.941
Syrians living outside camps	0.9	0.8	1.2	0.604	1.0	0.8	1.3	0.835
Palestinian living in a camp	0.4	0.2	0.9	0.020	0.7	0.3	1.5	0.317
Other	1.6	1.1	2.3	0.011	1.3	0.8	1.9	0.308

^a^Cigarette or Waterpipe: smoking either cigarettes or waterpipes, or both, versus non-smokers of either method; E-cigarettes: using e-cigarettes versus non-smokers, regardless of other used smoking methods.

^b^Hosmer–Lemeshow test: χ² = 6.42, df = 8, p = 0.6

^c^Hosmer–Lemeshow test: χ² = 5.40, df = 8, p = 0.71

Mother’s education level below a diploma was significantly associated with lower odds of e-cigarette use (OR = 0.8). Adolescents residing in the northern region of Jordan were significantly less likely to smoke cigarettes or waterpipe and use e-cigarettes, in contrast to those living in the central region of Jordan. Also, there was a distinct smoking pattern among different nationalities, where Palestinian camp refugees were significantly less likely than Jordanians to smoke cigarettes or waterpipe (OR = 0.4, 95% CI 0.2–0.9), but did not differ significantly in e-cigarette use. The Hosmer–Lemeshow test indicated adequate model fit for both logistic regression models (p > 0.05).

### Mental and psychosocial problems associated with smoking

In the multivariate analysis presented in Table 5, adolescents who reported smoking cigarettes or waterpipe had significantly higher scores and thus more separation anxiety symptoms, emotional symptoms, conduct problems, hyperactivity, peer relationship problems, overall difficulties, PTSD symptoms, and a higher risk for problematic Internet use. Also, smokers of cigarettes or waterpipe had significantly lower prosocial behavior scores (less social) and lower scores for health-related quality of life in all scales and subscales, and thus poorer quality of life in the total scale score, physical health summary score, psychosocial health summary score, emotional functioning, social functioning, and school functioning. The effect size was particularly large in HRQOL total and subscales, especially in school and emotional functioning.

**Table 5:** The association between mental and psychosocial problem scores (dependent variables) and adolescents’ use of smoking products (independent variables) ^b,c.^

Mental and psychosocial problems scales’ scores (Dependent variables) ^a^	Cigarette or waterpipe				E-cigarettes			
	Coefficient (B)	95% CI		P-value	Coefficient (B)	95% CI		P-value
**RCADS (Revised Child Anxiety and Depression Scale) total scales and subscales**								
Separation Anxiety Disorder	1.87	0.08	3.65	0.040	−0.78	−2.83	1.27	0.454
Generalized Anxiety Disorder	0.04	−1.33	1.41	0.953	−1.04	−2.62	0.53	0.195
Panic Disorder	0.42	−1.51	2.35	0.670	−1.30	−3.52	0.91	0.249
Social Phobia	0.03	−1.31	1.36	0.967	−0.19	−1.73	1.35	0.807
Obsessive-compulsive disorder	−0.07	−1.59	1.45	0.929	−0.83	−2.58	0.91	0.350
Major Depressive Disorder	−0.80	−2.45	0.85	0.343	0.20	−1.70	2.09	0.838
Total Anxiety	0.48	−1.11	2.07	0.550	−0.97	−2.79	0.86	0.300
**SDQ (Strengths and Difficulties Questionnaire) total scales and subscales**								
Emotional symptoms	0.62	0.35	0.90	<0.001	0.04	−0.28	0.36	0.804
Conduct problems	0.48	0.29	0.68	<0.001	0.54	0.32	0.76	<0.001
Hyperactivity	0.34	0.11	0.56	0.003	0.45	0.19	0.71	0.001
Peer relationship problems	0.63	0.42	0.85	<0.001	0.18	−0.06	0.43	0.145
Prosocial behavior	−0.34	−0.57	−0.11	0.004	−0.54	−0.81	−0.28	<0.001
Total difficulties	2.08	1.42	2.73	<0.001	1.21	0.46	1.97	0.002
Total CRIES-13 (Children’s Revised Impact of Event Scale-13) scale	3.14	1.48	4.79	<0.001	−0.20	−2.11	1.70	0.833
**PedsQL (Pediatric Quality of Life Inventory) total scales and subscales**								
Total HRQoL scale	−6.42	−8.40	−4.44	<0.001	−1.94	−4.22	0.34	0.095
Physical Health Summary	−5.54	−7.69	−3.38	<0.001	−0.97	−3.45	1.50	0.441
Psychosocial Health Summary	−6.96	−9.09	−4.84	<0.001	−2.40	−4.83	0.04	0.054
Emotional functioning	−8.42	−11.18	−5.66	<0.001	−1.69	−4.87	1.48	0.295
Social functioning	−4.69	−6.86	−2.52	<0.001	−1.30	−3.79	1.19	0.305
School functioning	−7.80	−10.32	−5.28	<0.001	−4.07	−6.96	−1.17	0.006
**Problematic Internet use scale**	1.36	0.69	2.03	<0.001	0.75	−0.02	1.52	0.057

^a^Higher scores indicate greater severity on RCADS, SDQ (except for the Prosocial Behavior subscale), CRIES-13, and the Problematic Internet Use Scale; Higher scores on PedsQL indicate better health-related quality of life and functioning.

^b^Cigarette or Waterpipe: smoking either cigarettes or waterpipes, or both, versus non-smokers of either method; E-cigarettes: using e-cigarettes versus non-smokers, regardless of other used smoking methods.

^c^Each model is adjusted for school classification, school type, gender, region, nationality, parents’ marital status, mother’s education, father’s education, and family income source.

Adolescents who reported using e-cigarettes had significantly higher conduct problems, hyperactivity symptoms, and total difficulties scores, as well as lower prosocial behavior and poorer school functioning. The strongest association was observed for school functioning.

## Discussion

### Smoking rates

In reviewing older studies and survey reports, despite differences in definitions of smoking and exclusion of e-cigarettes, consistent findings through the years underscore the persistently high prevalence of waterpipe smoking among youth in Jordan, particularly among males, and exceeding cigarette smoking rates. The results of the Global Youth Tobacco Survey (GYTS) show that in 2009, a greater percentage of 20.7% of youth aged 13–15 years were current waterpipe smokers (boys 27.1% vs. girls 15.6%), surpassing the 11.5% of current cigarette smokers (boys 17.4% vs. girls 6.6%) [20]. Similarly, in the 2014 GYTS, 26.7% of respondents were waterpipe smokers (boys 34.5% vs. girls 18.4%), surpassing the 11.4% of current cigarette smokers (boys 17.3% vs. girls 5.4%) [21]. Also, baseline data from a longitudinal study in 2008 further highlighted this trend among seventh-grade students in northern Jordan, with a larger percentage of 14.0% were current waterpipe smokers (boys 20.2% vs girls 7.5%), surpassing the 5.7% of current cigarette smokers (boys 9.0% vs girls 2.3%) [22]. It is important to acknowledge that variations in smoking prevalence estimates exist across studies because of the differences in the studied population, age group, inclusion criteria, and the definition of smoking across different forms.

In comparison to other Arab countries, Levantine nations such as Lebanon, Palestine, and Syria demonstrate similarly high rates of waterpipe smoking among youth, comparable to those observed in Jordan, which could be explained by the shared cultural dynamics and characteristics [23]. In contrast, Gulf and North African countries have relatively lower rates, potentially influenced by more conservative social norms and having waterpipe smoking less ingrained as a social practice compared to the Levant [23]. This underscores the influence of inherited and deeply rooted cultural traditions that shape adolescent waterpipe smoking behavior.

Overall, the persistently high rates of waterpipe smoking among youth in Jordan remain a critical public health concern, exacerbated by the rising popularity and use of e-cigarettes. Collectively, these trends contribute to an increasing burden of flavored tobacco product consumption, posing substantial threats to youth health and development. Flavored tobacco products have a strong appeal to youth due to their smell and taste, and in Jordan, the high rates could be attributed to additional factors, including the loosely enforced and updated regulations on flavored tobacco products, the greater social acceptability of waterpipe and e-cigarette smoking compared to manufactured cigarettes, the deep-rootedness of waterpipe smoking in social and cultural traditions, the use of social media for flavored tobacco advertising and sales despite existing prohibitions, the widespread misperception of their less harmful effects, and the view of flavored products as a cheaper alternative to smoking [9,10,24]. Also, there is a great availability of such products to young people through uncontrolled delivery services at home and in cafes in Jordan [25] and the positioning of waterpipe smoking as a daily experience or leisure activity fueled by the proliferation of specialty waterpipe cafes serving a diverse variety of fruit-mixed flavors of tobacco mixes.

### Smoking and sociodemographic factors

After accounting for other factors, sex was not associated with any smoking form, contrary to what is reported on the significantly higher prevalence in male adolescents in Arab countries for waterpipe from the pooled GYTS data [23] and for various forms and categories of smoking in Jordan [26]. This trend could be explained by the earlier nature of the previous studies, as many changes in Jordanian society have occurred, such as the changing population composition due to the reception of Syrian refugees and the dynamic change in gender norms and social acceptance of smoking, particularly for waterpipe and e-cigarettes among females, which is well established among adults and now seems to reach adolescents [10,27]. Other factors could include the probable reduced underreporting in surveys among females as social norms become more open. Another contributing factor may be the increasing influence of digital media, where adolescents of both sexes have broad access to tobacco-related content [28]. However, the sex impact on tobacco use could vary depending on the specific type of tobacco, and analyzing these categories separately might reveal more nuanced insights.

Age was a significant predictor of smoking of different types, with high odds of smoking cigarettes or waterpipe, and e-cigarettes with increasing age. This aligns with a longitudinal study from Irbid in Jordan among seventh-grade students, showing that waterpipe and cigarette smoking increased as students aged, with evidence that although waterpipe dominates early initiation, cigarette smoking rapidly escalates as adolescents age [29]. The increase in smoking behaviors with age among adolescents may be attributed to developmental characteristics typical of this life stage, including greater autonomy, broader social exposure, heightened curiosity, and experimentation. Additional factors such as media influence, peer pressure, and easier access to tobacco products further amplify this risk.

When looking at the difference between adolescents of different statuses within the country, there is little and conflicting evidence in the literature as to whether smoking rates are higher among refugees than among non-refugee adolescents. Our study showed that Syrians living in camps or outside camps had a relatively similar, non-significant likelihood of smoking compared to Jordanians, which could be due to some degree of acculturation and adoption of mainstream culture in Jordan, which may influence their smoking habits, as well as the already shared cultural dynamics and characteristics between Jordan and Syria. In contrast, Palestinians were significantly less likely to smoke cigarettes or waterpipe, which could be due to varying levels of health awareness and the effectiveness of public health campaigns targeting Palestinians in Jordan.

### Smoking and mental health outcomes

The results show that smoking among adolescents in Jordan is associated with various mental health problems and with a marked impact on quality of life, particularly evident among adolescents smoking cigarettes or waterpipe. These findings align with previous research that links adolescent cigarette smoking to conditions such as attention-deficit/hyperactivity disorder, oppositional defiant disorder, conduct disorder, anxiety, trauma exposure, and poor quality of life [30–33].

Studies on smoking among Jordanian adolescents have primarily focused on reporting prevalence rates, providing limited insights into associated psychosocial factors. However, one study examining Syrian refugees who smoke (both cigarettes and waterpipe) found no significant association with depression after adjustment for confounding factors [34], which is also shown in the current study. Regarding e-cigarette use, most of the associated mental health issues were linked to behavioral difficulties, with similar associations reported in previous studies [35,36].

However, the relationship between smoking behaviors and mental health issues, as well as the directionality of this association, remains complex and not fully understood. For example, some studies support smoking as a potential precursor to the development of depression [37,38]. Conversely, some studies [39,40] suggest that adolescents with existing depressive symptoms are at an increased risk of smoking. Overall, the findings reveal a link between smoking behaviors in the study population and mental health issues, highlighting the vulnerability of high-risk groups, such as adolescents with behavioral difficulties. These insights emphasize the need for targeted tobacco prevention programs that address underlying behavioral and psychological factors to effectively mitigate smoking-related risks.

### Policy Implications and Recommendations

First, it is crucial to continuously re-evaluate and monitor the implementation of existing policies while also incorporating updates and revisions informed by evidence, granular data analyses, and emerging smoking trends across different age groups in Jordan, particularly concerning flavored tobacco products. Global evidence indicates that flavored products increase the risk of nicotine addiction and traditional cigarette smoking among youth [41,42]. In response to this issue, strict regulatory updates to flavored tobacco product legislation are underway in several regions worldwide. For instance, Belgium adopted a broader approach by entirely banning disposable vapes, driven by their popularity among young people and the associated environmental risks [43] and Finland, on the other hand, has focused specifically on banning the characterizing flavors in e-cigarette liquids [44]. In Jordan, current laws impose restrictions on e-cigarettes, including age limits for purchasers set at 19 years and older and regulations on the composition and allowable flavors and additives [45]. However, challenges persist in the effective implementation and enforcement of these regulations. For instance, one study found that tobacco and electronic nicotine products were found to be sold in 69% of shops within a 150-meter radius of schools, and 14.3% were tobacco specialty stores, which is also closer than the legally mandated 250-meter limit [24]. It is also recommended to enforce strict regulations on the availability and delivery of waterpipe in restaurants and homes to adolescents, by placing restrictions or monitoring the credit processing of adolescents and robust age-verification procedures.

Second, given the immense influence that social media platforms have, they present an opportunity to be leveraged for raising awareness and promoting public health initiatives. A study analyzing content on TikTok between 2019 and 2022 found that the majority of English-language sampled e-cigarette-related posts portrayed e-cigarette use positively (97.7%), while only five posts portrayed e-cigarettes negatively (1.9%) and one post neutrally (0.4%); alarmingly, the e-cigarette positive videos received 98.7% of total views of all selected videos in the sample [46]. Thus, social media platforms have a wide reach, particularly among youth, making them powerful tools for awareness through targeted mass media campaigns and the strategic engagement of local and influential social media influencers. Population-based strategies, such as mass media campaigns, could be used to reach individuals with low socioeconomic status.

Third, it is recommended to implement school-based programs aimed at enhancing social competence and social influence, alongside education on the dangers of smoking. Preventive programs were found to be most effective when they are school-based or high-intensity programs, and when conducted by teachers or educators with proper training [47]. Based on evidence, school-based smoking prevention curricula should not solely focus on educating students about the dangers of smoking but also prioritize enhancing social competence and addressing the social influences that contribute to smoking behaviors [48]. Social competence interventions help adolescents refuse smoking offers by improving their general social competence and personal and social skills. Social influence interventions focus specifically on teaching adolescents skills for awareness of social influences that encourage substance use, and to resist tobacco offers, peer pressure, and high-risk situations that might persuade an adolescent directly or indirectly to smoke. Finally, it is recommended to address smoking and mental health altogether as part of a comprehensive smoking cessation approach, such as including stress management techniques and resilience-building practices.

## Strengths and limitations

This study provides updated national evidence on the prevalence of multiple forms of tobacco use among adolescents in Jordan, with a wide geographic scope and the inclusion of a broad age range of children and adolescents. However, the primary limitation of this study lies in its cross-sectional design. Cross-sectional studies offer only a snapshot at one point, making it impossible to establish temporal relationships. While this study explores the presence of an association, it does not definitively ascertain whether smoking constitutes a risk factor for mental health problems or vice versa. Consequently, we strongly advocate for the implementation of longitudinal study designs to provide evidence on the temporal relationship between smoking behavior and mental health problems, to understand the initiation age associated with specific smoking products, and to assess the impact of interventions and policies on smoking behavior over time. Non-response bias is another limitation that was addressed by oversampling to ensure adequate representation of all groups. Although the study used a stratified cluster sampling design, the analyses did not apply complex survey design–based methods. As a result, prevalence estimates should be interpreted as descriptive rather than population-representative, and variance estimates may be underestimated. Findings are therefore interpreted with caution, with emphasis placed on observed associations rather than precise prevalence estimates.

## Conclusion

Adolescent smoking in Jordan remains a pressing public health issue, with waterpipe use emerging as the most common form and increasing with age across different nationalities. Smoking, whether in the form of cigarettes, waterpipe, or e-cigarettes, was associated with increased vulnerability to a range of mental health issues and diminished health-related quality of life. Addressing this issue requires a multifaceted and evidence-based approach, including developing school-based prevention and control programs, incorporating social competence and social influence curricula, enforcing existing tobacco laws, and introducing updated regulations in response to emerging trends and evidence, particularly targeting flavored products. Furthermore, prevention and control strategies need to implement targeted interventions that address both the psychosocial roots of smoking and its consequences.

## Supporting information

S1 File Inclusivity-in-global-research-questionnaire. (DOCX)
